# Relationship of Community Mobility, Vital Space, and Faller Status in Older Adults

**DOI:** 10.3390/s24237651

**Published:** 2024-11-29

**Authors:** Diego Robles Cruz, Andrea Lira Belmar, Anthony Fleury, Méline Lam, Rossana M. Castro Andrade, Sebastián Puebla Quiñones, Carla Taramasco Toro

**Affiliations:** 1 Escuela de Ingeniería Civil Informática, Universidad de Valparaíso, Valparaíso 2361827, Chile; 2 Centro de Estudios del Movimiento Humano, Escuela de Kinesiología, Facultad de Salud y Odontología, Universidad Diego Portales, Santiago 8370076, Chile; 3 Instituto de Tecnología para la Innovación en Salud y Bienestar, Facultad de Ingeniería, Universidad Andrés Bello, Viña del Mar 2520000, Chile; electronico4.itisb@unab.cl (S.P.Q.); carla.taramasco@unab.cl (C.T.T.); 4 Center of Interdisciplinary Biomedical and Engineering Research for Health—MEDING Universidad de Valparaíso, Valparaíso 2520000, Chile; 5 IMT Nord Europe, Institut Mines Télécom, Centre for Digital Systems, 59650 Villeneuve d’Ascq, Francemeline.lam@etu.imt-nord-europe.fr (M.L.); 6 Group of Computer Networks, Software Engineering and Systems (GREat), Computer Science Department (DC), Federal University of Ceará (UFC), Campus do Pici, Bloco 910, Fortaleza 60440-900, Brazil; rossana@ufc.br; 7 Millennium Nucleus on Sociomedicine, Temuco 4811230, Chile

**Keywords:** fall risk, community mobility, gait patterns

## Abstract

Community mobility, encompassing both active (e.g., walking) and passive (e.g., driving) transport, plays a crucial role in maintaining autonomy and social interaction among older adults. This study aimed to quantify community mobility in older adults and explore the relationship between GPS- and accelerometer-derived metrics and fall risk. Methods: A total of 129 older adults, with and without a history of falls, were monitored over an 8 h period using GPS and accelerometer data. Three experimental conditions were evaluated: GPS data alone, accelerometer data alone, and a combination of both. Classification models, including Random Forest (RF), Support Vector Machines (SVMs), and K-Nearest Neighbors (KNN), were employed to classify participants based on their fall history. Results: For GPS data alone, RF achieved 74% accuracy, while SVM and KNN reached 67% and 62%, respectively. Using accelerometer data, RF achieved 95% accuracy, and both SVM and KNN achieved 90%. Combining GPS and accelerometer data improved model performance, with RF reaching 97% accuracy, SVM achieving 95%, and KNN 87%. Conclusion: The integration of GPS and accelerometer data significantly enhances the accuracy of distinguishing older adults with and without a history of falls. These findings highlight the potential of sensor-based approaches for accurate fall risk assessment in community-dwelling older adults.

## 1. Introduction

From a functional perspective, mobility can be described as the “capacity to move from one location to another independently” [1]. This capacity characterizes the activity and participation of an individual within the community and can be facilitated or hindered by a variety of factors, including physical abilities, cognitive function, psychosocial aspects, and the external environment in which individuals operate [2]. In the field of geriatric research, the mobility of older adults within the community is an area of increasing interest, given its profound impact on quality of life. Mobility is not only essential for accessing basic services but also has a positive impact on the mental and physical health of older adults [3].

The geographical area that an individual covers in their daily routine is referred to as “living space” [4] and has been considered a marker of physical and cognitive performance in community-dwelling older adults, as it reflects their capacity to move and interact within their environment, making it a critical determinant regarding quality of life [5]. It can be conceptualized as a series of zones radiating from the individual’s residence, representing varying degrees of mobility and independence. This concept provides a comprehensive measure of functional mobility over time, capturing not only the physical distance traveled but also the frequency and regularity of movement across various spaces [6] and includes travel by various means of transport [7]. The concept of living space originated in 1985 by May, Nayak, and Isaacs in their publication “The Living Space Diary: A Measure of Mobility in Older Adults at Home” [8], wherein they segmented living space into five concentric zones, starting with the bedroom and progressively extending to larger areas, including the rest of the house, surrounding grounds such as gardens, neighborhood blocks, and areas across busy streets. Years later, the “Living Space Questionnaire” was developed by Stalvey et al. in 1999 [6], followed by the “Living Space Assessment (LSA)” created by Baker et al. at the University of Alabama at Birmingham (UAB) in 2003, which measures mobility across five concentric zones: outside the bedroom, outside the house, within the neighborhood, outside the neighborhood but within the town, and outside the town, over the past four weeks [9]. The composite score ranges from 0 to 120, with higher scores indicative of greater mobility flexibility; thus, this measure can be seen as a predictor of morbidity, mortality, and healthcare utilization among the elderly population. Currently, studies have begun to utilize devices equipped with sensors to monitor and accurately measure living space, aiming to reflect the multidimensional nature of mobility [10]. These devices exhibit significant potential due to their objectivity, reduced burden on participants, and capacity to provide prolonged observations over time [11]. One selected instrument is GPS, which serves as a geographical indicator for describing an individual’s daily activities [12].

The living space of older adults serves not only as a physical location of residence, but also reflects the environmental complexity they encounter [13]. Community mobility within the living space is linked to risk behavior, particularly in relation to falls. The World Health Organization (WHO) defines a fall as an “event that causes a person to unintentionally rest on the ground, floor, or another lower level”.

The prevalence of falls among older adults is 26.5%, yet this figure may increase depending on the individual’s context [14]. The most vulnerable group comprises adults aged 65 and older, with the risk escalating as age increases, especially in the presence of signs of frailty [15]. According to recent studies, approximately one in four older adults experiences at least one fall each year. This not only increases the risk of physical injuries, but also contributes to a persistent fear of falling, which becomes a significant factor in increasing the likelihood of future falls. This fear negatively impacts mobility confidence, often disrupting gait, particularly in community settings where environmental challenges are common. The recurrent cycle of falls and fear highlights the importance of implementing preventive strategies that address both the physical and psychological factors associated with fall risk in older adults [16].

Walking, as an essential motor function in daily life, constitutes a common link to activities of daily living and undergoes changes as we age. Trips are one of the most frequent causes of falls during walking and are associated with increased variability [17] and decreased speed [18] of walking, as well as observed changes in cadence [19,20], variability in stride length, swing phase duration, and double support [20], all of which have been shown to be directly associated with an increased risk of falls. Regardless of whether the fall has resulted in injuries, this situation leads to a diminished functional capacity of the individual to perform daily activities, which consequently results in a reduction in mobility within the living space during the six months following the event [21]. A decrease in living space is associated with frailty, fear of falling, fall risk, and mortality [21,22], among other factors, which significantly impact the health and well-being of older adults. Mobility within the living space during aging is compromised not only by intrinsic physiological issues, such as balance and gait disorders, neurological diseases, cognitive impairments, muscular weakness, depression, obesity, polypharmacy, and urinary incontinence, but also by other age-related factors that contribute to reduced mobility and increased fall risk [23,24,25]. Demographic factors such as sex, age, low education level, and socioeconomic status [23,26] also play a significant role. When analyzed from a broader perspective, it encompasses other relevant multidimensional aspects, including those related to economic, material, and sociocultural factors [27].

Due to the increasing importance of understanding living space in gerontological research, this study aims to examine the relationship between fall risk and living space. We asked whether it is possible to identify older people with and without a history of falls by using metrics that represent living space. Our hypothesis is that participants who do not have a history of falls have characteristics of greater living space, so they must be differentiated.

## 2. Related Work

Research has increasingly focused on the relationship between physical activity and living space mobility in older adults. The association between objectively measured physical activity and lifespace mobility demonstrates how wearable sensors, such as accelerometers and GPS devices, can provide accurate quantification of lifespace mobility [28,29], which is why its use has gained importance in the evaluation of daily mobility among older adults. Studies on the subject reveal that higher levels of physical activity are strongly associated with greater mobility in the living space, which contributes to greater autonomy and the ability to interact with various environmental zones [28,30]. These findings underscore the importance of promoting physical activity to reduce frailty and improve quality of life while highlighting the utility of sensor-based methods for continuous, objective measurements of both activity and living space mobility [31,32].

A group of researchers explored how GPS data can reveal information about the relationship between daily mobility and well-being in community-dwelling older adults, demonstrating that higher levels of daily mobility, as measured via GPS, are positively correlated with improved mental health and well-being, emphasizing the connection between environmental engagement and overall quality of life [33]. This further strengthens the argument for encouraging greater mobility to improve the life outcomes of older adults. Additionally, wearable sensor technologies have expanded into living space mobility assessments. The development and validation of a mobility tracker prototype introduces a GPS recorder combined with an accelerometer to objectively monitor mobility patterns and activity levels in older adults. The device’s ability to track real-time movement and activity participation highlights its potential for geriatric research. Research highlights the importance of integrating sensor technologies into mobility studies, providing more accurate measures to inform interventions aimed at improving independence and reducing fall risks [34,35].

The dynamic relationship between physical activity and life-space mobility is further explored in objectively measured physical activity and changes in life-space mobility among older people. Using accelerometers and GPS devices, a study reveals that increased physical activity is associated with expanded living space, leading to greater functional independence. In contrast, decreases in activity levels result in a contraction of living space, emphasizing the need to maintain activity levels to preserve mobility and quality of life [36]. Wearable sensors have also been used to quantify real-world mobility patterns, as discussed in Quantifying Free-Living Community Mobility in Healthy Older Adults Using Wearable Sensors. In another study, researchers capture detailed information about how older adults move in their communities, revealing the advantages of wearable technologies to continuously monitor mobility without the limitations of laboratory environments [37]. These findings suggest that wearable sensors offer an unobtrusive but accurate means of assessing mobility, informing interventions aimed at preserving physical function in older populations [37].

A more comprehensive approach to understanding mobility is presented in Predictors of Real-Life Mobility in Community-Dwelling Older Adults, which explores both individual-level determinants and external factors, such as neighborhood walkability and social engagement. One study suggests that effective interventions to improve mobility must consider both personal and environmental influences, reflecting the complexity of mobility patterns in older adults [38]. On the other hand, another study reveals that older adults who carry out higher levels of physical activity tend to move in areas of larger living spaces, which is indicative of greater autonomy. This research emphasizes the importance of promoting physical activity as a means to maintain or improve the mobility of the living space, directly contributing to the well-being and independence of older adults [32].

Community Mobility Positioning System Indicators and Future Health Outcomes Among Older Adults shows that GPS-derived mobility indicators predict future health outcomes, such as physical decline and hospitalization rates. Research results demonstrated that the utility of monitoring community mobility can inform early interventions aimed at preserving health and improving outcomes in aging populations [39]. The use of GPS watches to monitor mobility in the living space highlights the relationship between this mobility and the health characteristics of older adults who use devices with global positioning systems.

Research links reduced mobility in the living space with negative health indicators, such as decreased physical function and increased risk of falls, showing the value of GPS technology in providing real-time assessments of mobility and health in older adults [40]. The challenges and opportunities of using GPS to track mobility patterns in older adults with dementia were explored in a GPS-based study to understand the outdoor mobility patterns of older adults with dementia [41]. This research highlights the reduced outdoor mobility of people with dementia and suggests that GPS-based monitoring can help healthcare providers assess decreased mobility and implement timely interventions.

On the other hand, one study pointed out discrepancies between objective GPS data and self-reported mobility measures. This study emphasizes the importance of integrating objective tools such as GPS to complement self-reported data, leading to a more comprehensive understanding of life-space mobility and its implications for health outcomes, particularly in relation to falls [42]. Generating GPS activity spaces that shed light on the mobility habits of older adults further investigates the value of GPS data in creating “activity spaces” that provide detailed information about how older adults navigate their environments. Other research emphasizes that these activity spaces offer more accurate real-world movement data compared to traditional self-reports, highlighting the importance of leveraging technology for mobility assessments [12].

Despite the increasing use of sensor-based technologies to assess mobility, research that explores a broader range of physical functions and overall functioning within the context of living space mobility remains lacking. Most studies tend to focus on isolated aspects, such as walking speed or step count, rather than investigating how multiple physical factors interact to influence mobility in various environments. Expanding the scope of these investigations is essential to gain a more complete understanding of how living space mobility is affected by the complex interplay of physical and environmental factors. Additionally, integrating data from different sources, such as GPS and accelerometers, provides a more holistic view of mobility patterns. GPS data enable precise tracking of geographic movement and distance, while accelerometers provide detailed information on activity intensity and gait quality. This combination is crucial for understanding life-space mobility and its relationship to falls, as it offers insights into the spatial and physical dimensions of mobility, leading to improved fall-prevention strategies. However, there is still a significant gap in the literature on the combined use of sensor-based data to explore how various physical and environmental factors interact in shaping living space mobility. Our study seeks to fill this gap by integrating data from GPS, accelerometers, and other functional measures to provide a more complete perspective of mobility in older adults. By addressing this gap, our work seeks to contribute to the deepening of how these factors jointly influence fall risk, complementing with new knowledge that can inform more effective fall prevention interventions and mobility support strategies.

## 3. Materials and Methods

### 3.1. Participant Recruitment and Eligibility Criteria

A total of 129 older adults from the Metropolitan Region of Santiago, Chile, were included regardless of their socioeconomic level (see Table 1 for the description of the dataset). The recruitment process was adjusted to ethical guidelines, granting each participant written informed consent previously approved by the Scientific Bioethics Committee of the Central University of Chile (reference project 48/2022). Participants were recruited over a six-month period (August 2023–January 2024) through a stratified recruitment process in primary care centers and local communities. Recruitment efforts included advertisements in health centers and community networks inviting men and women aged 65 years and older to participate. Eligibility criteria required that participants were free of fatigue or breathing difficulties at the time of assessment. Older adults with chronic conditions such as hypertension or diabetes were included as long as their conditions were under medical control. However, people with cognitive impairment that could make it difficult to understand informed consent or follow simple instructions were excluded. The sampling method used was non-probabilistic convenience sampling, intended to guarantee a representative sample of diverse origins, socioeconomic levels, and health states.

Falls were identified through a combination of self-reported history and clinical assessment. During the baseline interview, participants reported any falls they had experienced in the past 12 months. When available, medical records were reviewed to corroborate reported falls. A fall was considered to be that defined by the WHO in 2021 as an “event which results in a person coming to rest inadvertently on the ground or floor or other lower level”. Based on this definition, participants were classified into two groups: those who had a history of falls and those who did not.

### 3.2. Use of Smartphones for Mobility Monitoring

**Figure 1:**
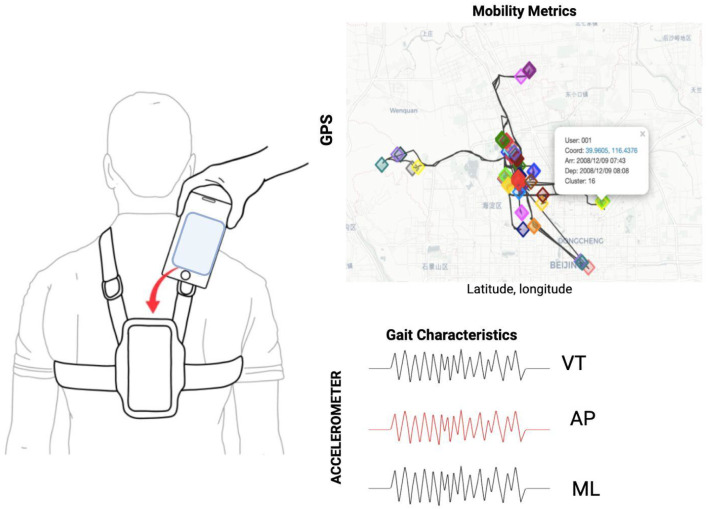
Phone location (**left**), GPS trajectories (rendering taken from Scikit-mobility (**right**) and accelerometer signals).

Mobility within the community was monitored using two systems: global positioning system (GPS) and using an accelerometer, both incorporated into a smartphone. The GPS provided estimates of life-space mobility metrics, while the accelerometer captured spatiotemporal parameters of gait and motor control. Smartphones, equipped with sensors such as accelerometers, gyroscopes, magnetometers, and GPS, serve as valuable, low-cost, and accessible tools for assessing mobility in older adults. The accuracy and reliability of smartphones equipped with MEMS-based inertial sensors, including the accelerometer and gyroscope found in devices such as the Samsung Galaxy A14, have been demonstrated in controlled laboratory tests. Studies have validated the performance of these sensors in various motion tracking applications, showing that they provide comparable accuracy to more advanced systems like motion capture devices [43]. Studies show that the accelerometer and gyroscope in modern smartphones provide valid and reliable data for estimating motion with comparable accuracy to standard systems, such as the Vicon MX motion capture system [44]. The studies show acceptable accuracy in measuring movement and orientation, making them viable for non-clinical applications such as gait analysis and fall prediction [44,45]. The GPS sampling rate was set to one data point per minute, which provided sufficient detail to track spatial movement patterns. The accelerometer, on the other hand, recorded data at a rate of 50 Hz (50 data points per second), which is adequate for capturing detailed gait dynamics. The mobile phone was securely placed on the participant’s torso, specifically between the T5 and T10 vertebral levels, using an adjustable harness to ensure stability and continuous contact with the individual’s body throughout the assessment (see Figure 1). This anatomical location was chosen due to its proximity to the center of mass of the body, providing a more representative measure of trunk balance and control during movement. Placing the phone in this area also ensures that the device does not interfere with the body’s natural movements during the assessment, providing comfort to the subject without compromising the accuracy of the data. This level of precision in device placement is critical for replicability by ensuring that the data collected accurately reflect the participant’s mobility and motor control. Each participant was monitored for approximately 8 h until they went to sleep.

The measurements began at a community center located within the participants’ residential municipality. From this starting point, each participant moved freely toward their home or other destinations, using their preferred mode of transportation (walking, by car, by bus, etc.). This approach allowed us to capture mobility in real-life conditions, reflecting their daily routines without imposing restrictions on their routes or activities.

### 3.3. Life-Space Mobility

Participants were instructed to follow their usual daily routines on the day they were assessed for community mobility. This approach was taken to ensure that the mobility data collected reflected authentic real-world behaviors and movement patterns. Although the observation period was limited to a single day, this method aimed to capture the typical mobility of participants within their community environment. To maintain the validity of the data, the assessments were rescheduled in case of unfavorable weather conditions, such as rain, which could have influenced the participants’ behavior. This precaution helped minimize deviations from their usual activities, ensuring a more accurate reflection of their mobility patterns. Participants were thoroughly briefed to adhere to their normal schedules, avoiding any variations, such as adding or omitting activities, to ensure that their daily living space and interactions were authentically captured. By emphasizing the importance of maintaining their usual routines, this study sought to provide a realistic assessment of life-space mobility in older adults within their natural community environments.

### 3.4. GPS Data Processing

Python’s Scikit-Mobility library was used to perform a detailed analysis of participants’ movement trajectories based on GPS (latitude and longitude) time series data. This tool allowed the visualization of movement routes, the identification of stopping places throughout the day, and the detection of the home location of each subject. Additionally, the time spent in each identified location was calculated. This information was then used to analyze acceleration patterns outside the home, providing a comprehensive understanding of subjects’ mobility and spatiotemporal behaviors within their daily environments.

Scikit-Mobility is an advanced Python library designed specifically for mobility data manipulation and analysis. It allows the study of various aspects of human movement through a specialized set of tools [46]. One of its key features is the TrajDataFrame class, which extends the functionality of Pandas DataFrame by integrating spatial and temporal coordinates. This allows specific operations on trajectories, such as filtering by time or location or calculating distances between points. The library also includes visualization functions for mapping trajectories, making it easier to interpret mobility patterns. In addition, Scikit-Mobility incorporates algorithms to detect locations where people stay for significant periods, allowing points of interest or repetitive behavior patterns to be identified. As shown in Figure 1, diamonds of different colors represent various stages in an individual’s trajectory, providing information about mobility behaviors and daily routines.

#### Daily Mobility Plot

**Figure 2:**
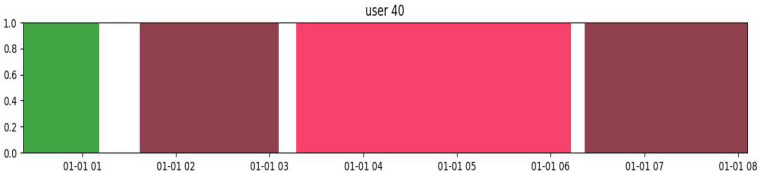
Participant’s daily mobility visualization with distinct areas of activity and movement range. Note: The green, brown, and white rectangles represent time spent in various locations outside the home, while the pink rectangle indicates the time spent inside the participant’s home.

The daily mobility of each participant was visually represented by a series of colored bars, each of them corresponding to different places visited throughout the day (see Figure 2). These color-coded bars illustrate both the duration and sequence of stays at various points of interest, offering a clear overview of participants’ daily movement patterns, including time thresholds and geographic radius considerations. The beginning of each bar indicates the time of arrival at a location, while the end marks the departure, creating a chronological visualization of mobility across geographic spaces. This graph highlights not only the frequency and regularity of visits to specific places, but also the spatial and temporal distribution of each individual’s activities throughout the day. In Figure 2, the pink rectangle represents the time spent inside the participant’s house, while the other colored rectangles (brown, white, green) mean different places visited. From this visualization, we identified the spatiotemporal windows corresponding to the periods in which participants moved outside their homes. To accurately generate these stays, several key parameters were defined. First, the stop radius factor, which determines the distance within which a location is classified as a “stay”; secondly, the minimum duration necessary to define a stop, which in this study was set at 15 min; and finally, the spatial radius, which represents the distance used to group nearby points. In this analysis, we used a radius of 100 m and a minimum stop duration of 15 min to identify significant stays. These parameters were carefully selected to capture relevant movements without introducing unnecessary noise on short trips or brief stops. The choice of a 100 m radius is justified by its proven suitability for capturing daily movements, especially in densely populated urban areas. This value strikes a balance between precision and functionality in detecting points of interest within mobility patterns [46,47]. Similarly, the 15 min stop duration was selected to filter out brief interruptions and focus on the most meaningful activities. Previous studies have employed similar thresholds to capture significant stays that reflect important human behaviors, such as visiting a commercial establishment or resting in a park [48,49].

### 3.5. Location Clustering

The location clustering function within the library enables the grouping of locations based on their spatial proximity, making it useful for identifying areas with high mobility density or geographic patterns of interest. This is achieved using the Density-Based Spatial Clustering of Applications with Noise (DBSCAN) algorithm, which clusters points based on density. Key parameters for this process include the maximum distance, which defines the maximum distance in kilometers for considering points within the same cluster, and the minimum number of samples required to form a cluster.

### 3.6. Mobility Metrics

The following life-space mobility variables were calculated using GPS data to provide a comprehensive view of spatial and temporal mobility patterns among the participants. These metrics offer valuable insights into both the geographical extent and behavioral characteristics of movement, both of which are for understanding life-space mobility.

- Activity Space (km^2^):
  This refers to the geographical area in which a person conducts their daily activities or regularly travels [11]. It is represented by the Minimum Convex Polygon (MCP), which is the smallest convex shape that encloses all the GPS points. The area of the MCP is calculated as
  $$A_{MCP}=\frac{1}{2}\sum_{i=1}^{n}\left(x_{i}y_{i+1}-x_{i+1}y_{i}\right)$$
  where $\left(x_{i},y_{i}\right)$ are the coordinates of the GPS points. In the code, this was converted from square meters to square kilometers.
- SDE Compactness: SDE compactness measures how concentrated or dispersed a person’s movements are within their activity space. This measure can provide information on the efficiency and distribution of mobility in a specific area [50].
  This is computed using the Standard Deviational Ellipse (SDE), which represents the spread of movement. Compactness is calculated as
  $$\mathrm{SDE}\;\mathrm{Compactness}=\frac{4\pi A_{SDE}}{P_{MCP}^{2}}$$
  where $A_{SDE}$ is the area of the ellipse and $P_{MCP}$ is the perimeter of the minimum convex polygon.
- Minimum Convex Polygon: The term minimum convex polygon refers to the geometric shape that minimally encloses all points, representing the mobility of older adults within their activity space. This polygon can be used to delineate the maximum area covered by daily activities of an older adult [4]. Its area, $A_{MCP}$, is computed as described in the Section 4.1.1.
- Number of Visited Places: Refers to the count of distinct locations visited by an older adult within a specified time period, which can be an indicator of their mobility and social activity [11]. It is calculated by identifying clusters of GPS points using the DBSCAN algorithm and counting the number of clusters.
  $$N_{places}=\sum_{i=1}^{n}\mathrm{unique}\_\mathrm{locations}\left(cluster_{i}\right)$$
- Time Outside of Home (hours): The total number of hours that an older adult spends outside their habitual residence can be a significant indicator of their level of activity and community participation [4]. The time spent outside the home is calculated by summing the time intervals during which the individual is located outside their home. If $t_{out_{i}}$ is the time outside for each visit:
  $$T_{outside}=\sum_{i=1}^{n}t_{out_{i}}$$
- Maximum Distance (km): The term maximum distance refers to the farthest distance an older adult has traveled from their habitual residence as part of their daily mobility [11]. It is calculated using the geodesic distance formula between the home location and the farthest point, as follows:
  $$D_{max}=\max_{i}\left(d_{geodesic}\left(home,\left(x_{i},y_{i}\right)\right)\right)$$
- Straight-Line Distance: This is the sum of the straight-line distances between consecutive points [51], calculated as
  $$D_{total}=\sum_{i=1}^{n-1}d_{geodesic}\left(P_{i},P_{i+1}\right)$$
  where $d_{geodesic}\left(P_{i},P_{i+1}\right)$ represents the geodesic distance between consecutive points.
- Turning Radius: This metric provides insights into the smoothness and continuity of an individual’s movement patterns. A smaller turning radius may indicate frequent changes in direction, often associated with navigation through confined spaces or complex environments. In contrast, a larger turning radius typically suggests smoother, more direct paths, often seen in open or less complex environments. In studies of older adults, a smaller turning radius could indicate cautious or hesitant movement, which might be linked to fear of falling or difficulties in spatial navigation [52]. It is calculated by evaluating the angles between consecutive trip segments and the radius of curvature, as follows:
  $$R_{turn}=\frac{l}{2\sin(\theta )}$$
  where *l* is the distance between two points, and θ is the angle of turning.
- K Radius of Gyration: A measure used to describe the dispersion of an individual’s movements from a central point, usually their home or another significant location. It calculates the average distance of all the points visited from this central location, providing an understanding of the extent of an individual’s mobility within their community. A larger radius of gyration suggests that the individual regularly moves over a broader area, indicating higher mobility and possibly greater independence [53]. It is computed as
  $$R_{g}=\sqrt{\frac{1}{n}\sum_{i=1}^{n}{\left(d_{i}-\bar{d}\right)}^{2}}$$
  where $d_{i}$ is the distance of the *i*-th point from the center of mass (usually the home location) and $\bar{d}$ is the average distance.

### 3.7. Accelerometer Data Processing

A Butterworth band-pass filter was applied to the acceleration signals on all three axes of the accelerometer, with low and high cut-off frequencies of 0.5 Hz and 20 Hz, respectively. This filter is effective in eliminating high-frequency noise while retaining the signal components relevant to human gait.

Subsequently, the ‘find_peaks’ function from ‘scipy.signal’ was used to detect peaks in the filtered signals. A minimum distance between consecutive peaks was set, calculated as half of the sampling frequency (fs/2) to avoid false detection caused by small fluctuations in the signal. Additionally, a minimum peak height threshold was defined, set to 0.5 to reduce the detection of peaks originating from residual noise. Peak identification enabled the calculation of various gait characteristics such as the number of steps, cadence, mean step time, step time variability, regularity, and step symmetry.

To exclusively detect and analyze the gait sections within the 8 h accelerometer data, specific inclusion criteria were implemented to identify walking episodes and exclude movements not relevant to gait analysis. First, a speed threshold of less than 5 km/h was set to exclude movements conducted via other modes of transportation, such as cars, buses, or public transit, thus focusing the analysis on gait activities.

Additionally, to ensure the continuity of the gait episodes, the acceleration signal was required to contain at least 30 consecutive peaks, representing detected steps. With the established threshold, brief or incomplete movements that do not reflect sustained gait patterns were excluded. On the other hand, the duration of the gait sections varied across participants, as it depended on their unique mobility patterns and movements throughout the study period. For each gait variable (such as cadence, mean step time, step time variability, regularity, and step symmetry) the average of all walking records that met these criteria was calculated and analyzed.

#### Gait Characteristics (Accelerometer Metrics)

- Number of Steps: Represents the total number of steps detected in the acceleration signal. It is calculated by identifying the peaks in the filtered signal, as follows:
  $$N_{\mathrm{steps}}=N_{\mathrm{peaks}}$$
  where $N_{\mathrm{peaks}}$ is the number of peaks detected in the signal.
- Mean Step Time: The mean time between consecutive steps is calculated as
  $$T_{\mathrm{mean}}=\frac{1}{N-1}\sum_{i=1}^{N-1}\left(t_{i+1}-t_{i}\right)$$
  where $t_{i}$ is the time of the *i*-th peak and *N* is the total number of steps.
- Step Time Variability: Calculated as the coefficient of variation,
  which is the ratio of the standard deviation to the mean step time, as follows:
  $$CV_{\mathrm{step}\mathrm{time}}=\frac{\sigma_{T}}{T_{\mathrm{mean}}}$$
  where $\sigma_{T}$ is the standard deviation of the step times.
- Step Regularity: A measure of the consistency of step times, calculated as the inverse of the standard deviation of the step time, as follows:
  $${\mathrm{Regularity}}_{\mathrm{step}}=\frac{1}{\sigma_{T}}$$
- Step Symmetry: Assesses the equality of alternating step times. It is calculated as  
  $$\mathrm{Symmetry}=1-\frac{1}{N-1}\sum_{i=1}^{N-1}\left|\frac{T_{i+1}-T_{i}}{T_{\mathrm{mean}}}\right|$$
  where $T_{i}$ is the step time and $T_{\mathrm{mean}}$ is the mean step time.
- Walking Speed: Calculated as the total distance traveled (number of steps multiplied by the average step length) divided by the total recording time, as follows:
  $$v=\frac{N_{\mathrm{steps}}\times L_{\mathrm{step}}}{T_{\mathrm{total}}}$$
  where $L_{\mathrm{step}}$ is the average step length and $T_{\mathrm{total}}$ is the total recording time.
- RMS Acceleration: The root mean square (RMS) of the acceleration signal across different axes is calculated as
  $$a_{\mathrm{RMS}}=\sqrt{\frac{1}{N}\sum_{i=1}^{N}a_{i}^{2}}$$
  where $a_{i}$ is the acceleration at time *i* and *N* is the number of samples.
- Harmonic Ratio: The ratio between the energies of even and odd harmonics in the acceleration signal is calculated as
  $$HR=\frac{\sum_{\mathrm{even}}E_{\mathrm{harmonics}}}{\sum_{\mathrm{odd}}E_{\mathrm{harmonics}}}$$
  where $E_{\mathrm{harmonics}}$ represents the energy of the harmonics in the signal.
- Entropy: A measure that quantifies the degree of disorder or randomness in the signal, calculated using a histogram of the acceleration values. Entropy is defined as
  $$H=-\sum_{i=1}^{n}p_{i}\log\left(p_{i}\right)$$
  where $p_{i}$ is the probability of the *i*-th bin in the histogram and *n* is the number of bins.

In this study, an average step length of 0.7 m was used as a constant for all participants. This value is widely recognized in the literature as a standard approximation for step length in older adults during regular walking conditions. Research has shown that the average step length for healthy adults typically ranges between 0.65 and 0.75 m depending on factors such as height, gait speed, and age [54,55]. Given the variability in step length due to individual characteristics, using 0.7 m as an average provides a reasonable estimate for analyzing walking patterns in older populations. Studies have also demonstrated that fixed step length values are commonly employed in gait analysis when direct measurement is not feasible or when individual-specific data are unavailable [56,57]. This approach ensures consistency in the estimation of walking speed and total distance traveled, allowing for a reliable comparison of gait parameters across participants.

Step count calculation is a key element for gait pattern analysis and was based on the detection of peaks in the vertical acceleration signal. The use of spike detection in acceleration signals is a well-established technique in gait analysis. Previous studies have shown that identifying peaks in the vertical acceleration signal is a reliable and valid method for detecting steps, particularly in free-living conditions and with wearable devices such as smartphones and accelerometers [58,59]. While it may seem simplistic, peak detection is very effective due to the repetitive and cyclical nature of walking, where each step generates a distinct peak in the acceleration signal. This method has been widely used in clinical and research settings as it provides accurate step count estimates, especially when combined with filtering techniques such as the Butterworth filter to reduce noise and eliminate false detections [60,61]. Furthermore, other step-derived variables such as step time variability, regularity, and symmetry, are also well-supported in the literature as indicators of gait stability and fall risk. These variables, derived from step intervals and accelerometer data, have been shown to correlate with balance control and fall risk in older adults [54,62]. Therefore, by combining a standardized step length with peak detection for step counting, and subsequently deriving additional gait metrics, this study employs a validated approach that strikes a balance between simplicity, practicality, and accuracy in both controlled and free-living environments. Additionally, the use of the inverse of the standard deviation of step time as a measure of step regularity is also well-supported in the literature. Studies have shown that this metric provides a reliable and simple quantification of step rhythm coherence, reflecting variability in the gait cycle. For example, several studies [54,62] used the inverse of the standard deviation of step time to evaluate gait stability, which demonstrated its usefulness in identifying individuals at higher risk of falls due to irregular stepping patterns. Although step regularity is often derived using autocorrelation procedures, particularly in studies that focus on variations between strides and the periodicity of gait signals [63], the inverse of the standard deviation remains a widely accepted and simpler, computationally efficient, and interpretable alternative, especially in large data sets or free-living conditions. This method allows a balance between simplicity and reliability, providing a meaningful measure of gait consistency that correlates with other indicators of gait stability, such as symmetry and gait variability.

### 3.8. Mobility and Gait Metrics

The inclusion of both life-space mobility and gait characteristics as features in this study is essential for providing a comprehensive analysis of mobility patterns in older adults and their association with fall risk. Life-space mobility metrics, such as time spent outside the home, number of places visited, radius of gyration, and maximum distance traveled, capture an individual’s spatial behavior and their interaction with the environment. These metrics are particularly important because reduced life-space mobility has been linked to increased frailty, social isolation, and a higher likelihood of falls [64,65]. By analyzing these variables, we can better understand how participants’ engagement with their surroundings influences their physical activity levels and overall fall risk. In addition, gait characteristics such as step count, walking speed, stride regularity, and step symmetry provide critical insights into the biomechanical aspects of mobility. Gait has been extensively studied as a predictor of falls, with irregularities in gait patterns often indicating underlying motor or balance impairments [62,66]. Features such as step time variability and RMS acceleration help capture not only the consistency of movement but also the stability of the gait cycle, which are crucial indicators of an individual’s functional mobility and fall risk [67,68]. By combining both life-space mobility and gait characteristics, this study is able to capture both the environmental and physical dimensions of mobility, offering a more holistic approach to fall risk prediction. This comprehensive set of features allows for a better understanding of the interplay between spatial behavior and motor control, thus improving the accuracy of the models used to classify fall risk in older adults.

### 3.9. Classification Between Fallers and Non-Fallers

Using mobility metrics and machine learning algorithms, we assessed the ability to differentiate behavioral patterns between older adults with and without a history of falls in the past year. To achieve this, we employed Support Vector Machines (SVMs), Random Forest (RF), and K-Nearest Neighbors (KNN) algorithms. The selection of these algorithms was based on their complementary strengths in handling diverse data types and their proven effectiveness in classification tasks, particularly within the domain of gait analysis and fall risk prediction.

SVM is renowned for its robustness in high-dimensional feature spaces and performs particularly well with small sample sizes, making it an ideal choice for our dataset, which consists of a limited number of participants relative to the feature space [69,70]. Furthermore, studies have demonstrated that SVM classifiers exhibit high generalizability in automatic fall detection, achieving up to 93% accuracy in distinguishing falls from normal daily activities [71]. KNN, on the other hand, is a simple yet powerful instance-based learning algorithm that performs well in scenarios where the decision boundaries are complex or non-linear. KNN’s ability to adapt locally to the structure of the data makes it a natural choice when the relationship between features and labels may vary across different regions of the feature space [72,73]. KNN has shown high performance in classifying gait patterns, with reported accuracy rates of up to 94% [74].

Finally, Random Forest (RF) was chosen for its strong performance in handling noisy and high-dimensional data, thanks to its ensemble nature and ability to mitigate overfitting through bootstrapping and random feature selection [75]. RF has been widely applied in fall detection and gait analysis studies, consistently demonstrating high accuracy. In a recent study, RF achieved 91.3% accuracy in predicting fall risk using gait data [76].

By selecting these three algorithms, we aimed to leverage their respective advantages to improve the robustness and accuracy of our fall risk classification model. To implement the algorithms, the Python Scikit-learn library was used, which provides simple and efficient tools for data analysis and modeling, including classification, regression, clustering, and dimensionality reduction algorithms.

In this study, three classification models were implemented: Random Forest, K-Nearest Neighbors, and Support Vector Machines, to differentiate between the groups of “people who have experienced falls” and “people who have not experienced falls”. To optimize the performance of each model, an exhaustive hyperparameter search was conducted using GridSearchCV. For Random Forest, combinations of hyperparameters were explored, including the number of trees (n_estimators) set at 100, 200, and 300; maximum tree depths (max_depth) of 2 and 3; minimum number of samples required to split an internal node (min_samples_split) of 2 and 5; minimum number of samples required at a leaf node (min_samples_leaf) of 3 and 4; and the number of features to consider when looking for the best split (max_features) as ‘sqrt’ and ‘log2’. For the K-Nearest Neighbors model, values of n_neighbors from 1 to 10 were evaluated, with weight functions (weights) ‘uniform’ and ‘distance’, and distance metrics ‘Euclidean’ and ‘Manhattan’. Regarding Support Vector Machines, values of the penalty parameter C of 0.1, 1, and 10 were tested; values of gamma of 1, 0.1, and 0.01; and different kernel functions: ‘rbf’, ‘poly’, and ‘sigmoid’. Cross-validation was performed using Leave-One-Out Cross-Validation (LOOCV), and the f1_macro metric was used to evaluate performance during hyperparameter tuning. The data were divided into training and test sets in a proportion of 70% and 30%, respectively, applying standard normalization and missing value imputation through a pipeline that included SimpleImputer and StandardScaler. To evaluate the final performance of the models, classification reports and ROC curves were generated, using the area under the curve (AUC) as the main metric.

The ROC curves were generated using the test datasets following the split into training and test sets, without retraining the models on the samples used in the ROC curve analysis. This methodology ensures that the AUC calculation accurately reflects the model’s performance on the validation set.

## 4. Results

We compared two groups: individuals without a history of falls (no fall events throughout their lifetime) and those with a history of falls (individuals who experienced one or more falls in the past 12 months). The following graphs and tables present the relationship between the reported fall history over the past year and the various estimated metrics. To assess the distribution of the variables, the Shapiro–Wilk test was conducted. The results indicated that all variables exhibited non-parametric distributions, with *p*-values less than 0.05 for both the no-fall group and the fall group. Due to the non-parametric nature of the data, the Mann–Whitney U test was subsequently applied to compare the groups across each variable. The Mann–Whitney U test is suitable for non-parametric data as it does not rely on the assumption of normality, providing a robust method to evaluate the statistical significance of differences between the two groups.

### 4.1. Inferential Statistics Results of Spatial Mobility (GPS Metrics)

#### 4.1.1. Activity Space

The analysis of Activity Space (km²) revealed significant differences between the non-faller and faller groups. The median activity space for the non-faller group was 0.85 km² (IQR = 0.84), while the faller group showed a median of 0.61 km² (IQR = 1.09). The Mann–Whitney U test indicated a significant difference between the groups with a *p* = 0.012 (U = 2538.00).

**Figure 3:**
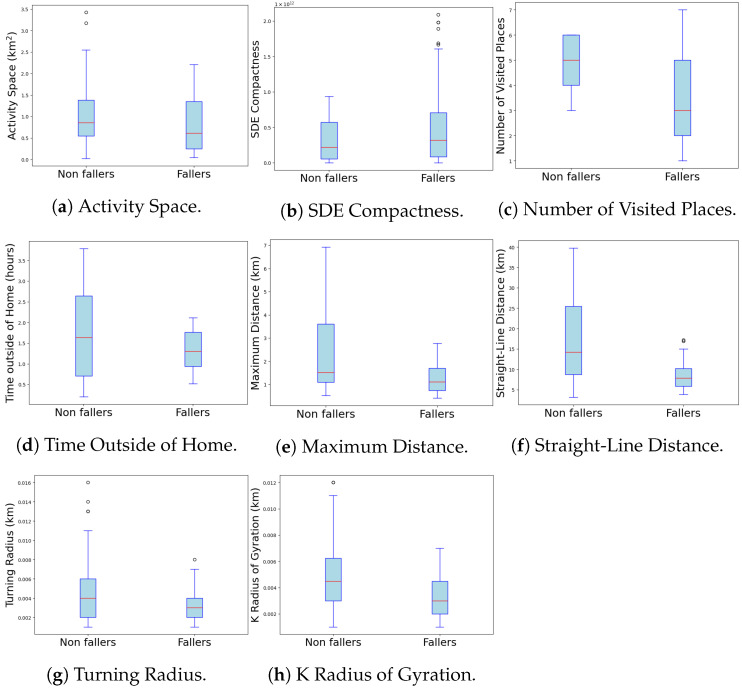
Differences in spatial mobility variables between non-fallers and fallers groups (GPS metrics).

These findings suggest that subjects without a history of falls tend to have a larger activity space compared to those with a history of falls, which may imply greater mobility and physical capability in the non-faller group (Figure 3a and Table 2). The substantial difference observed in Activity Space (km²) for both the non-faller and faller groups can be attributed to the considerable variability in mobility and physical capabilities among individuals in each group (Figure 3a). This variance suggests that, while the average activity space highlights general trends—such as larger spaces in non-fallers, implying better mobility—the high standard deviation reflects significant diversity in individual mobility levels. Factors such as age, health status, and lifestyle differences among participants likely contribute to this variability, reflecting the complex nature of mobility in older adults.

#### 4.1.2. Maximum Distance

The Maximum Distance (km) variable shows a significant difference between the non-faller and faller groups. Non-faller individuals traveled a median Maximum Distance of 1.52 km (IQR = 2.51) while faller individuals traveled an median of 1.11 km (IQR = 0.96). The Mann–Whitney U test indicated that this difference is statistically significant, with a *p* < 0.001 (U = 2412.00). These findings suggest that non-faller individuals are able to travel greater distances compared to those with a history of falls, which may be related to greater physical endurance and mobility (Figure 3e and Table 2).

#### 4.1.3. Straight-Line Distance

The Straight-Line Distance (km) variable shows significant differences between the non-faller and faller groups. The median straight-line distance for non-faller individuals is median: 14.23 km (IQR = 16.79), while for faller individuals, the median straight-line distance is 7.87 km (IQR = 4.32). The Mann–Whitney U test yielded *p* < 0.001, indicating that the difference is statistically significant (U = 2378.00). These results suggest that non-faller individuals tend to move over longer straight-line distances compared to those with a history of falls, potentially reflecting better navigational abilities and overall physical health (Figure 3f and Table 2).

### 4.2. Non-Significant Differences in Mobility Space Parameters

Among the variables examined, SDE Compactness, Number of Visited Places, Turning Radius, K Radius of Gyration, and Total Time Away from Home did not show significant differences between the two groups. Specifically, SDE Compactness (Figure 3b), which measures the compactness of the spatial distribution of the subject’s movement, had a *p*-value of *p* = 0.791, indicating no significant difference between fallers and non-fallers. Similarly, the Number of Visited Places (Figure 3c), which reflects the variety of locations frequented by the subject, also did not differ significantly between the groups, with a *p* = 0.79. The Turning Radius (Figure 3g), a parameter that describes the radius of turns made during movement, showed a *p*-value of *p* = 0.087, further suggesting no significant variation between fallers and non-fallers. Lastly, the K Radius of Gyration (Figure 3h), which represents the dispersion of the subject’s movement from a central point, yielded a *p*-value of p=0.032, indicating a lack of significant difference in this parameter as well. The variable Total Time Away from Home (hours) also did not show statistically significant differences between the non-faller and faller groups. The non-faller group spent a median of 1.63 h away from home (IQR = 1.94), while the faller group spent a median of 1.3 h (IQR = 0.83). A p-value of *p* = 0.066 suggests that this observed difference is not statistically significant (Figure 3d and Table 2).

### 4.3. Inferential Statistics Results of Gait (Accelerometer Metrics)

#### 4.3.1. Number of Steps

**Figure 4:**
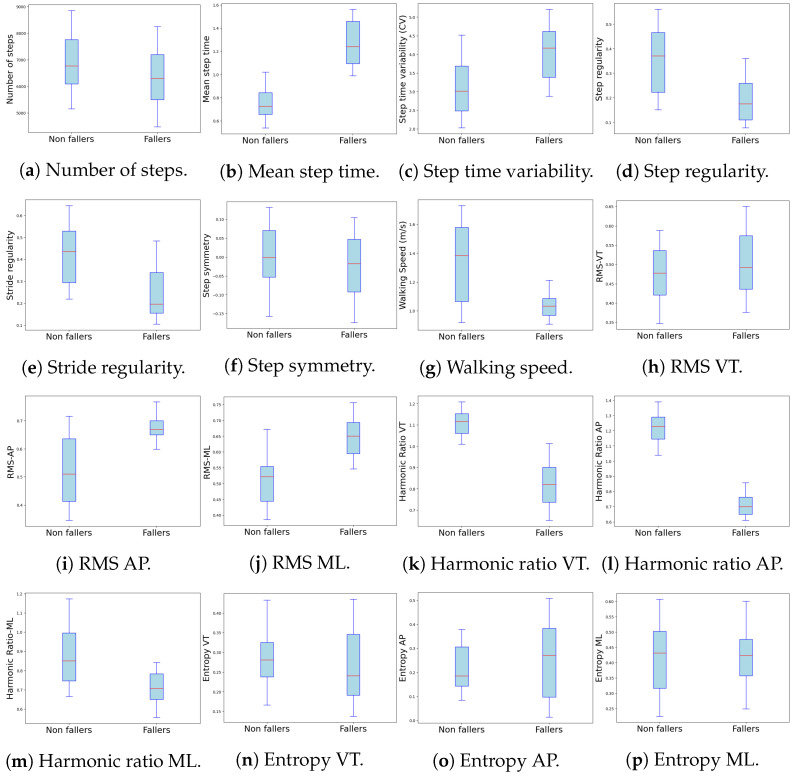
Differences in gait results between non-fallers and fallers groups (accelerometer metrics).

The difference in the number of steps taken during a day of recording did not show significant differences between the groups (*p* = 0.63), despite the nearly one thousand-step difference between them. The non-faller group took a median of 6770 steps (IQR = 1667), while the faller group took a median of 6298 steps (IQR = 1692) (see Figure 4a and Table 3).

#### 4.3.2. Step Time

The group with a history of falls exhibited a significantly longer median of step time of 1.24 s (IQR = 0.36), compared to the shorter median of step time of 0.73 s (IQR = 0.19) observed in the group without a history of falls (*p* < 0.001). This difference suggests a slower gait pace in the faller group, which may be due to excessive caution or impaired motor control, reflecting a reduced ability to maintain a consistent walking rhythm (see Figure 4b and Table 3).

#### 4.3.3. Step Time Variability (Coefficient of Variation, CV)

The group with a history of falls exhibited a significantly higher coefficient of variation with a median of 4.17 (IQR = 1.23), compared to 3.01 (IQR = 1.21) in the group without a history of falls with a *p*-value *p* < 0.001. This difference indicates greater instability in step time, suggesting a less consistent gait and a higher propensity for falls. In contrast, the lower coefficient of variation observed in the group without a history of falls reflects a more stable walking rhythm. This significant difference in step time variability reinforces the idea that rhythm instability is more pronounced in individuals with a history of falls, thereby increasing their risk of future falls (see Figure 4c and Table 3).

#### 4.3.4. Step Regularity

The significant difference in step regularity was *p* < 0.001, which highlights greater gait consistency in the group without a history of falls, with a median value of 0.37 (IQR = 0.24), compared to 0.18 (IQR = 0.15) in the group with a history of falls. This consistency is crucial for maintaining stability and preventing falls (see Figure 4d and Table 3).

#### 4.3.5. Stride Regularity

The significant differences observed (*p* < 0.001) suggest that individuals with a history of falls exhibit a less regular stride pattern, with a median value of 0.2 (IQR = 0.19), compared to 0.44 (IQR = 0.24) in the group without a history of falls. Reduced stride regularity may result in difficulties adapting to changes or irregularities in the terrain, thereby further increasing the risk of falls (see Figure 4e and Table 3).

#### 4.3.6. Step Symmetry

Although no significant differences were found in step symmetry (*p* = 0.600), with median values of −0.002 (IQR = 0.124) for the group without a history of falls and −0.018 (IQR = 0.14) for the group with a history of falls, symmetry remains an important aspect of gait. While not statistically significant in this study, asymmetries may reflect compensations or adaptations that could increase the risk of falls (see Figure 4f and Table 3).

#### 4.3.7. Gait Speed

The group with a history of falls walks at a significantly slower speed with a *p* < 0.001, with a median speed of 1.0335 m/s (IQR = 0.12), compared to 1.382 m/s (IQR = 0.51) in the group without a history of falls. This reduced speed may be a strategy to prevent falls. However, it could also limit the ability to respond to perturbations, thereby increasing the risk of falls (see Figure 4g and Table 3).

#### 4.3.8. Root Mean Square of Trunk Acceleration

The RMS represents the average magnitude of acceleration in different planes of the body (anteroposterior, vertical, mediolateral). Higher RMS indicates greater oscillations in trunk movement. These oscillations may reflect a lower capacity for motor control, greater instability, and a constant need for postural adjustments to maintain balance while walking. This parameter has been validated in the scientific literature as an indicator of alterations in gait control and a reliable predictor of the risk of falls in older adults [61,77]. For all three planes of movement, the group with a history of falls exhibited significantly higher RMS values compared to the group without a history of falls (*p* < 0.001) for all three planes). Specifically, the group with a history of falls showed a median RMS of 0.67 (IQR = 0.05) in the anteroposterior (AP) plane, compared to 0.51 (IQR = 0.224) in the non-faller group; an RMS of 0.478 (IQR = 0.115) in the vertical (VT) plane, compared to 0.492 (IQR = 0.139) in the faller group; and an RMS of 0.65 (IQR = 0.09) in the mediolateral (ML) plane, compared to 0.52 (IQR = 0.11) in the non-faller group. These findings suggest that individuals with a history of falls experience greater difficulty maintaining smooth, controlled movements across all planes, potentially increasing their risk of future falls (see Figure 4h–j and Table 3).

#### 4.3.9. Harmonic Ratio Across the Three Axes of Movement

The group with a history of falls exhibited significantly lower harmonic ratios (*p* < 0.001) across all three planes of movement. A lower harmonic ratio may indicate difficulties in maintaining smooth and consistent motion suggesting less coordinated and less efficient movement characterized by a less stable, less rhythmic, and thus less safe gait. In contrast, the higher harmonic ratios observed in the group without a history of falls indicate more coordinated and efficient movement, reflecting better stability and safer gait patterns.

Specifically, the HR in the AP plane for the group with a history of falls was a median of 0.701 (IQR = 0.114), compared to 1.228 (IQR = 0.144) in the non-faller group. In the VT plane, median HR was 0.82 (IQR = 0.165) for the faller group, compared to 1.12 (IQR = 0.09) in the non-faller group. In the ML plane, the median HR was 0.71 (IQR = 0.134) for the faller group, compared to 0.852 (IQR = 0.25) in the non-faller group. These findings underscore the reduced stability and coordination in the gait of individuals with a history of falls, further highlighting their increased risk (see Figure 4k–m and Table 3).

#### 4.3.10. Entropy Across the Three Axes of Movement

The entropy variable did not show significant differences between the groups, suggesting that the complexity of movement is similar in both groups. Specifically, the *p*-values for the VT axis (*p* = 0.139), the AP axis (*p* = 0.516), and the ML axis (*p* = 0.851) indicate no significant variation in entropy.

In the AP axis, the group with a history of falls had a median of entropy value of 0.271 (IQR = 0.286), compared to 0.1855 (IQR = 0.162) in the non-faller group. For the VT axis, the median of entropy value was 0.241 (IQR = 0.15) in the faller group, compared to 0.281 (IQR = 0.09) in the non-faller group. Similarly, for the ML axis, the entropy was 0.423 (IQR = 0.118) in the faller group, compared to 0.43 (IQR = 0.186) in the non-faller group. These findings indicate that the overall complexity of movement, as measured by entropy, does not differ significantly between individuals with and without a history of falls (see Figure 4n–p and Table 3).

### 4.4. Classification Algorithms Results

#### 4.4.1. GPS Data Classification Results

**Figure 5:**
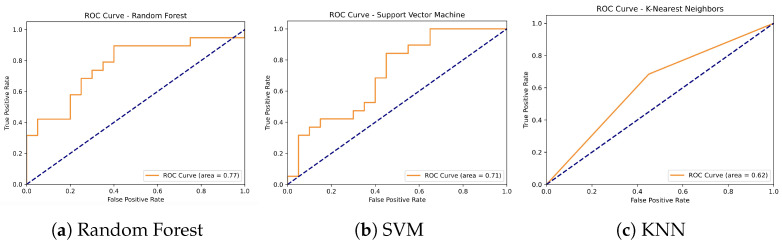
ROC curves for classification of fallers and non-fallers using GPS data.

The classification performance of three machine learning algorithms—Random Forest, Support Vector Machine (SVM), and K-Nearest Neighbors (KNN)—was evaluated using GPS-derived data to distinguish between non-fallers and fallers (see Table 4 and Figure 5). The Random Forest model achieved an accuracy of 74%, with a precision of 0.86 for non-fallers and 0.68 for fallers, and recall values of 0.60 and 0.89 for non-fallers and fallers, respectively. The SVM model demonstrated an accuracy of 67%, with precision values of 0.73 for non-fallers and 0.62 for fallers, and recall values of 0.55 for non-fallers and 0.79 for fallers. In contrast, the KNN model achieved an accuracy of 62%, with precision values of 0.65 for non-fallers and 0.59 for fallers, and recall values of 0.55 for non-fallers and 0.68 for fallers. Overall, Random Forest outperformed both SVM and KNN in terms of precision, recall, and F1-score, indicating its stronger ability to classify non-fallers and fallers based on GPS data.

To calculate the accuracy and consistency of the classification models, the Leave-One-Out Cross-Validation (LOOCV) method was used, ensuring that each observation was employed for validation, which is particularly useful in datasets with limited size, as in this study. From the cross-validation, we estimated the standard deviation (SD) of the accuracy results, which reflected the variability in the performance of the classifiers across different iterations. For the Random Forest model, the SD was 0.48, while both SVM and KNN showed an SD of 0.45. These values indicated that all three classifiers exhibited moderate variability in their performance across different runs. Despite the similarity in standard deviation, Random Forest achieved a higher average accuracy, making it the most robust and effective model for classifying participants as fallers or non-fallers based on GPS data (see Table 4).

#### 4.4.2. Accelerometer Data Classification Results

The classification performance of three machine learning algorithms—Random Forest, Support Vector Machine (SVM), and K-Nearest Neighbors (KNN)—was evaluated using accelerometer-derived data to distinguish between non-fallers and fallers. The Random Forest model achieved the highest overall accuracy of 95%, with a precision of 0.91 for non-fallers and 1.00 for fallers, and a recall of 1.00 for non-fallers and 0.89 for fallers. The SVM model demonstrated an accuracy of 90%, with precision values of 0.94 for non-fallers and 0.86 for fallers, and recall values of 0.85 for non-fallers and 0.95 for fallers. Similarly, the KNN model achieved an accuracy of 90%, with a precision of 0.90 for non-fallers and 0.89 for fallers, and recall values of 0.90 for non-fallers and 0.89 for fallers. Overall, the Random Forest model outperformed the SVM and KNN models, providing the most balanced classification results in terms of precision, recall, and F1-score.

**Figure 6:**
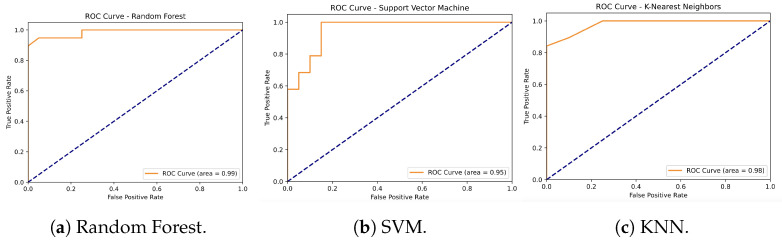
ROC curves for classification of fallers and non-fallers using accelerometer data.

In terms of standard deviation (SD) for the accuracy results, the Random Forest model had a standard deviation of 0.181, indicating relatively low variability in its performance across different runs. The SVM model, with a standard deviation of 0.231, showed slightly more variability compared to Random Forest, but still maintained consistent results. The KNN model exhibited the same standard deviation as Random Forest, 0.181, indicating a similarly low level of variability in performance. These results suggest that while all models demonstrated moderate variability, Random Forest and KNN offered more consistent accuracy, whereas SVM showed slightly more fluctuation in performance. Despite the differences in SD, all three models performed with moderate consistency when using accelerometer data (see Table 5 and Figure 6).

#### 4.4.3. GPS and Accelerometer Data Classification Results

**Figure 7:**
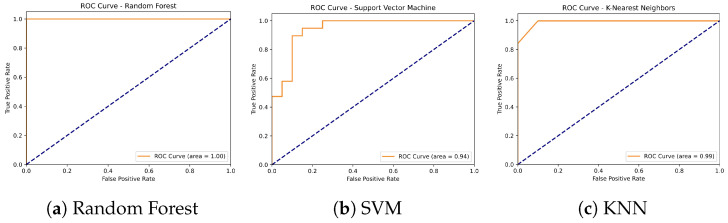
ROC curves for classification of fallers and non-fallers using combined GPS and accelerometer data.

The performance of three machine learning algorithms—Random Forest, Support Vector Machine (SVM), and K-Nearest Neighbors (KNN)—was compared for the classification of non-fallers and fallers using combined GPS and accelerometer data (see Table 6 and Figure 7). The Random Forest model achieved an overall accuracy of 97%, with a precision of 1.00 and recall of 0.95 for the non-faller group, and a precision of 0.95 and recall of 1.00 for the faller group. The SVM model also demonstrated a high accuracy of 95%, with perfect precision for non-fallers (1.00) and slightly lower precision for fallers (0.90), and recall values of 0.90 for non-fallers and 1.00 for fallers. In contrast, the KNN model showed a lower performance, with an accuracy of 87%, a precision of 0.94 for non-fallers and 0.82 for fallers, and recall values of 0.80 for non-fallers and 0.95 for fallers. Across all algorithms, Random Forest and SVM consistently outperformed KNN in terms of precision, recall, and F1-scores for both groups, indicating their superior ability to distinguish between fallers and non-fallers using the combined dataset.

In terms of standard deviation (SD) for the accuracy results, the Random Forest model had the lowest variability, with an SD of 0.15, indicated a more consistent performance across different runs or data subsets. The SVM model showed slightly higher variability, with an SD of 0.23, suggested moderate fluctuation in its classification performance. The KNN model, with the highest SD of 0.25, demonstrated more variability compared to Random Forest and SVM. This suggests that while Random Forest provided the most consistent and accurate results, KNN exhibited more fluctuation in its performance. Despite this, all models demonstrated moderate variability, with Random Forest being the most reliable in terms of consistency.

## 5. Discussion

The primary objective of this study was to assess whether machine learning models could effectively distinguish between older adults with and without a history of falls using mobility metrics derived from GPS and accelerometer data. By employing three machine learning algorithms (Random Forest, Support Vector Machine, and K-Nearest Neighbors) we sought to explore how life-space mobility and gait patterns could predict fall risk in older adults. This study addressed a gap in the literature by demonstrating that the combination of sensor-based metrics, such as GPS and accelerometers, enhances classification performance compared to using individual data sources alone.

Our results indicated significant differences in several key metrics, underscoring the relevance of these measures for fall risk prediction. For instance, the “Activity Space” metric showed substantial differences between non-fallers and fallers (see Table 2 and Figure 3a), as did the “Maximum Distance Traveled” (see Figure 3e). These findings suggest that non-fallers tend to have greater mobility within their life space, potentially reflecting better functional mobility. Similarly, gait metrics, such as mean step time and step time variability, revealed slower and more variable gait patterns in the group of individuals who experienced falls, consistent with a less stable gait in those with a history of falls (see Figure 4b,c).

Furthermore, models that combined GPS and accelerometer data outperformed those based on either data source alone. The Random Forest model achieved the highest accuracy at 97%, closely followed by SVM at 95%, while KNN showed lower performance with an accuracy of 87% (see Table 6 and Figure 7). These findings are consistent with previous research [78], which demonstrated that machine learning models like Random Forest and SVM are highly effective in predicting fall risk when using wearable sensors for gait and movement analysis; similarly, [79] found that combining accelerometer data with machine learning algorithms improved fall risk classification accuracy in older adults.

In contrast, models based solely on GPS data yielded lower accuracies, with Random Forest achieving 74% and SVM and KNN reaching 67% and 62%, respectively (see Table 4 and Figure 5). While these results suggest that GPS data alone provide valuable information about life-space mobility, it is the combination of GPS and accelerometer data that produces the most robust predictions, reinforcing the importance of integrating both data sources for fall risk assessment. Additionally, classification models based solely on accelerometer data also showed strong results: Random Forest again achieved the highest accuracy at 95%, followed by SVM and KNN with 90%. These findings demonstrate the utility of gait characteristics captured by accelerometers for predicting fall risk, while also highlighting that adding GPS metrics offers greater refinement and enhances the predictive power of the models (see Table 5 and Figure 6).

Previous research has often relied heavily on accelerometer data to classify fallers and non-fallers. For example, a study [80] demonstrated that accelerometer-based models could achieve high accuracy in detecting fall risk, with results reaching up to 90% precision. However, this study did not incorporate life-space mobility metrics, such as activity space derived from GPS data. This limits the approach to movement characteristics captured by accelerometers without considering spatial mobility patterns that reflect the overall functional capacity of older adults. In contrast, this study adds to the existing literature by demonstrating that the combination of life-space mobility metrics with gait characteristics results in significantly improved classification outcomes. The inclusion of GPS data, as demonstrated in this work, adds an important dimension to understanding fall risk, particularly by capturing variations in daily movement patterns that reflect an individual’s functional capacity. Another study [81] emphasized that older adults with restricted life-space mobility are at greater risk of falling, corroborating the findings of this study, which showed that fallers exhibited significantly smaller activity spaces and shorter maximum distances traveled compared to non-fallers. Integrating these GPS-derived metrics into machine learning models, as demonstrated here, strengthens the case for using life-space variables to assess fall risk in community-dwelling older adults.

Using GPS metrics in combination with gait characteristics provides a more comprehensive assessment of fall risk. Non-fallers exhibited larger life-space metrics, including greater activity space and maximum distance traveled, which aligns with previous studies suggesting that reduced mobility is associated with a higher risk of falls. Additionally, gait metrics such as step time variability and harmonic ratio showed significant differences between fallers and non-fallers, reinforcing the importance of stable and rhythmic movement patterns in fall prevention. These findings are consistent with those of Weiss et al. (2013) [78], who found that algorithms such as Random Forest and SVM, when applied to wearable sensor data (e.g., accelerometers), were highly effective in predicting fall risk among older adults. However, this study demonstrates that combining GPS and accelerometer data further improves prediction outcomes.

Interestingly, while some metrics showed significant differences between fallers and non-fallers, others, such as step symmetry, did not. This may suggest that certain gait characteristics, like symmetry, may not be as relevant for distinguishing fallers from non-fallers or could reflect limitations in the sample size or sensitivity of measurement tools. This finding highlights the need for further studies exploring which gait characteristics are most predictive of fall risk. Moreover, differences in recall rates between fallers and non-fallers suggest that while the models are good at identifying non-fallers, there is room for improvement in accurately detecting fallers. This observation aligns with the findings obtained in a study [79] in which it was noted that although accelerometer data with machine learning algorithms, improved fall risk classification accuracy, accurately detecting fallers remains challenging, particularly in individuals with more restricted life-space mobility.

Despite the significant findings, this study presents several limitations that should be considered when interpreting the results. One major limitation is the sample size. Although this study included 129 older adults, this number may not be large enough to capture the full variability of gait and mobility metrics within this population. A larger sample size could improve the generalizability of the results and allow for more detailed analyses, such as stratification by age groups or comorbidities, which was not feasible in this study due to the limited number of subjects. Additionally, the mobility and gait data were collected over a single day of observation. While participants were asked to maintain their normal daily routine, this approach may not adequately capture fluctuations in mobility patterns that occur over the course of a week or different seasons. Future studies should consider collecting data over more extended periods to better reflect variability in older adults’ mobility and their environment. Another consideration is the use of a fixed step length estimation (0.7 m) to calculate walking speed and total distance traveled. While this value is consistent with previous studies and provides a reasonable approximation for the population studied, individual step length was not measured for each participant, which may have introduced estimation errors in the walking speed analysis. In future research, it would be beneficial to incorporate personalized step length measurements or use more advanced sensors capable of estimating this value in real time. Moreover, the use of peak detection in accelerometer signals for step counting, while widely accepted in the field of biomechanics, may not adequately capture steps occurring in unusual conditions or during abrupt changes in direction. This could limit the precision in identifying irregular gait patterns in individuals at high risk of falls. A combination of detection methods, such as using gyroscopes alongside accelerometers, could enhance accuracy in future research.

The use of machine learning algorithms, although powerful, also comes with certain limitations. While Random Forest offers high classification accuracy, the use of this algorithm implies the need for a larger amount of data to improve its generalization capability. Since machine learning algorithms tend to be sensitive to the quality and quantity of data, future research should include larger and more diverse datasets to validate the findings of this study. Additionally, hyperparameter tuning techniques were not explored, which could have further optimized the models’ performance. Moreover, this study did not address multicollinearity in the mobility and gait metrics used to train the classification models. The presence of multicollinearity could affect the interpretation of the individual contributions of variables in the model and bias the results. Future studies should employ techniques such as Principal Component Analysis (PCA) or the removal of highly correlated variables to enhance model stability and interpretability. Finally, the definition of the group of non-fallers, based exclusively on the absence of fall events in the 12 months preceding the assessment, represents another limitation. While this definition is consistent with the literature and allows for an initial classification, it does not guarantee that individuals classified as “non-faller” will not experience a fall shortly after measurement. Given that falls can be unpredictable events, the fact that a person did not fall in the previous year does not eliminate the possibility of a future fall. This introduces uncertainty into the participant categorization, and future studies should consider longitudinal follow-up to more accurately capture fall risk over the long term, providing a better understanding of the factors that may predispose an individual to fall in the period following the initial assessment.

Turning back to the findings, this study makes several contributions to the field of mobility and fall risk prediction in older adults by integrating novel metrics from GPS and accelerometer data. First, our work expands on previous research by providing a detailed analysis of life-space mobility and gait patterns using the combined use of these two sensor modalities. By tracking community mobility with GPS and examining gait characteristics using accelerometer data, we were able to develop a more comprehensive model for predicting fall risk. This combination of spatial and temporal data has not been widely explored in the existing literature and allows for a richer understanding of how environmental factors and motor control intersect in the context of aging. Additionally, our study presents strong classification results for distinguishing between older adults with and without a history of falls, using machine learning algorithms such as Random Forest, K-Nearest Neighbors, and Support Vector Machines. Lastly, future research could focus on extending the temporal scope of this study by lengthening the observation period beyond a single day and collecting data across various environmental contexts. This approach would provide a more nuanced understanding of how mobility behaviors fluctuate over time and under different environmental conditions. Additionally, the findings of this study could inspire the development of intelligent wearable devices capable of real-time monitoring of gait and movement patterns, sending immediate alerts to caregivers or family members when high fall-risk patterns are detected. Such devices could employ advanced anomaly detection algorithms to identify sudden changes in the user’s mobility or stability. Furthermore, these data could be integrated into personalized rehabilitation programs, dynamically adjusting physical and therapeutic activities according to the user’s abilities and monitoring their progress. This would enable more effective and safer rehabilitation for individuals who have fallen or are at high risk of falling. In the long term, implementing AI-based predictive models could also aid in estimating future fall risk, facilitating early preventive interventions such as targeted exercise programs, home modifications, or adjustments in medical treatments to mitigate risk factors.

## 6. Conclusions

The incorporation of mobility metrics based on GPS and accelerometry has enabled a comprehensive analysis of the daily movement patterns of elderly people within the community. These features provide valuable insights into physical capabilities and their relationship to movement, particularly in relation to fall risk. Overall, this study highlights the potential of combining GPS accelerometry technology with machine learning algorithms to advance our understanding of mobility patterns in older adults and to develop proactive measures for fall prevention. 

## Figures and Tables

**Table 1:** Subjects enrolled in this study.

	Age (years)		Weight (kg)		Height (cm)	
	Non-Faller	Faller	Non-Faller	Faller	Non-Faller	Faller
Valid Sample Size	65	64	65	64	65	64
Mean	72.68	71.03	68.27	70.36	151.63	156.82
Std. Deviation	6.93	7.25	11.84	11.83	28.51	8.79

**Table 2:** Comparison of mobility space parameters between fallers and non-fallers using the Mann–Whitney U test (GPS metrics).

Parameter	*p*
Activity Space (km²)	0.012
SDE Compactness	0.791
Number of Visited Places	0.079
Time Outside of Home (hours)	0.066
Maximum Distance (km)	<0.001
K Radius of Gyration (km)	0.032
Turning Radius	0.087
Straight-Line Distance	<0.001

Note. Mann–Whitney U test was used for all comparisons.

**Table 3:** Comparison of gait parameters between fallers and non-fallers using the Mann–Whitney U test (accelerometer metrics).

Parameter	*p*
Number of steps	0.063
Mean step time	<0.001
Step time variability (CV)	<0.001
Stride regularity	<0.001
Step regularity	<0.001
Step symmetry	0.443
Walking speed (m/s)	<0.001
RMS-ML	<0.001
RMS-AP	<0.001
RMS-VT	0.018
Harmonic Ratio-ML	<0.001
Harmonic Ratio-VT	<0.001
Harmonic Ratio-AP	<0.001
Entropy-ML	0.996
Entropy-VT	0.383
Entropy-AP	0.209

Note. Mann–Whitney U test was used for all comparisons.

**Table 4:** Comparison of algorithms for non-faller and faller classification using GPS data.

Model	Precision		Recall		F1-Score		Acc	SD
	Non-Faller	Faller	Non-Faller	Faller	Non-Faller	Faller		
Random Forest.	0.86	0.68	0.60	0.89	0.71	0.77	0.74	0.48
SVM.	0.73	0.62	0.55	0.79	0.63	0.70	0.67	0.45
KNN.	0.65	0.59	0.55	0.68	0.59	0.63	0.62	0.45

Acc: Accuracy; SD: Standard Deviation.

**Table 5:** Comparison of algorithms for non-faller and faller classification using accelerometer data.

Model	Precision		Recall		F1-Score		Acc	SD
	Non-Faller	Faller	Non-Faller	Faller	Non-Faller	Faller		
Random Forest	0.91	1.00	1.00	0.89	0.95	0.94	0.95	0.1815
SVM	0.94	0.86	0.85	0.95	0.89	0.9	0.90	0.2315
KNN	0.90	0.89	0.90	0.89	0.9	0.89	0.90	0.1815

Acc: Accuracy; SD: Standard Deviation.

**Table 6:** Comparison of algorithms for non-faller and faller classification using gps and accelerometer data.

Model	Precision		Recall		F1-Score		Acc	SD
	Non-Faller	Faller	Non-Faller	Faller	Non-Faller	Faller		
Random Forest.	1.0	0.95	0.95	1.0	0.97	0.97	0.97	0.15
SVM.	1.00	0.90	0.90	1.00	0.95	0.95	0.95	0.23
KNN.	0.94	0.82	0.80	0.95	0.86	0.88	0.87	0.25

Acc: Accuracy; SD: Standard Deviation.

## Data Availability

The data supporting this research are available upon request. To request access to the data, please contact: diego.robles@postgrado.uv.cl.

## Abbreviations

The following abbreviations are used in this manuscript:

EP	Elderly people
GPS	Global positioning system
MCP	Minimum convex polygons
SVM	Support Vector Machine
RF	Random Forest
KNN	K-Nearest Neighbors Classification
SD	Standard deviation
LOCCV	Leave-One-Out Cross-Validation
Acc	Accuracy
